# Drug-Induced Anaphylaxis: National Database Analysis

**DOI:** 10.3390/ph17010090

**Published:** 2024-01-09

**Authors:** Olga Butranova, Sergey Zyryanov, Anastasia Gorbacheva, Irina Asetskaya, Vitaly Polivanov

**Affiliations:** 1 Department of General and Clinical Pharmacology, Peoples’ Friendship University of Russia Named after Patrice Lumumba (RUDN), 6 Miklukho-Maklaya St., 117198 Moscow, Russia; zyryanov-sk@rudn.ru (S.Z.); 1052212124@rudn.ru (A.G.); asetskaya-il@rudn.ru (I.A.); 2 Moscow City Health Department, City Clinical Hospital No. 24, State Budgetary Institution of Healthcare of the City of Moscow, Pistzovaya Srt. 10, 127015 Moscow, Russia; 3 Pharmacovigilance Center, Information and Methodological Center for Expert Evaluation, Record and Analysis of Circulation of Medical Products under the Federal Service for Surveillance in Healthcare, 4-1 Slavyanskaya Square, 109074 Moscow, Russia; pvit74@gmail.com

**Keywords:** drug-induced allergy, anaphylaxis, antibiotics, local anesthetics, cyclooxygenase (COX)-inhibitors

## Abstract

(1) Background: National health system databases represent an important source of information about the epidemiology of adverse drug reactions including drug-induced allergy and anaphylaxis. Analysis of such databases may enhance the knowledge of healthcare professionals regarding the problem of drug-induced anaphylaxis. (2) Methods: A retrospective descriptive analysis was carried out of spontaneous reports (SRs) with data on drug-induced anaphylaxis (SRsAs) extracted from the Russian National Pharmacovigilance database (analyzed period 2 April 2019–21 June 2023). The percentage of SRsAs among SRs of drug-induced allergy (SRsDIAs) was calculated, as well as of pediatric, elderly, and fatal SrsAs. Drugs involved in anaphylaxis were assessed among total SRsAs, pediatric, and elderly SRsAs, and among fatal SRsAs. Demographic parameters of patients were assessed. (3) Results: SRsAs were reported in 8.3% of SRsDIAs (2304/27,727), the mean age of patients was 48.2 ± 15.8 years, and females accounted for 53.2% of cases. The main causative groups of drugs were antibacterials (ABs) for systemic use (44.6%), local anesthetics (20.0%), and cyclooxygenase (COX) inhibitors (10.1%). Fatal SRsAs were reported in 9.5% (218/2304) of cases, the mean age of patients was 48.0 ± 16.7 years, and females accounted for 56.4% of cases. Pediatric SRsAs accounted for 3.9% of pediatric SRsDIAs and 5.8% of all SRsAs, with a mean age of 11.8 ± 4.5 years, and females acccounted for 51.9% of cases. Elderly SRsAs accounted for 2% of elderly SRsDIAs and 2.8% of all SRsAs, and the mean age was 73.0 ± 5.3 years, and females accounted for 43.5% of cases. ABs caused 40% of SRsAs in the elderly, 42.9% in children, and 50% of fatal SRsAs. (4) Conclusions: Our study revealed a relatively high proportion of anaphylaxis among SRs of drug-induced allergy. ABs were the most prevalent causative agents, especially in fatal SRsAs.

## 1. Introduction

The prevalence of severe allergic reactions, including anaphylaxis, is increasing worldwide with the most frequent elicitor groups including food, insect venom, and drugs [1,2,3,4]. Drug allergy may affect up to one-third of patients in emergency departments [5]. The prevalence of self-reported drug allergy was estimated in a systematic review and meta-analysis of 53 studies (*n* = 26,306) to be 8.3% (range across studies 0.7–38.5%) [6]. Anaphylaxis is one of the most severe forms of allergy, representing an acute, potentially fatal reaction. The World Allergy Organization Anaphylaxis Guidance 2020 [1] defined anaphylaxis as “a serious systemic hypersensitivity reaction that is usually rapid in onset and may cause death” and stated that “severe anaphylaxis is characterized by potentially life-threatening compromise in airway, breathing and/or the circulation, and may occur without typical skin features or circulatory shock being present”.

A cross-sectional study based on a screening of medical records (from January 2015 to August 2017) identified the prevalence of anaphylaxis among emergency department admissions at the level of 0.00026%, and the pediatric population (age 1–16 years) was the most affected (60.9%). Anaphylaxis was triggered by drugs in 17.4% [7]. A retrospective 10-year study of practice in a tertiary hospital revealed drugs to be the most common cause of anaphylaxis in an emergency department (33%) [8]. A nearly two-fold difference between the percentages of drug-induced anaphylaxis reported in the given studies may be explained by the time factor (in the first study [7], less than 3-year period was analyzed, from January 2015 to August 2017, while in the second [8], a 10-year period was analyzed, from January 2007 to December 2016), and also by the type of analyzed population (in the first study [7], both children and adults were analyzed, in the second [8], only adults). An analysis of electronic health records from a large United States healthcare system described the epidemiology of drug-induced hypersensitivity reactions (HSRs) and found that they were reported in 13.8% (377,474 out of 2.7 million patients), of which 53.1% were immediate type reactions and 46.9% were delayed reactions [9]. 

Anaphylactic reactions are among the most dangerous immediate type HSRs, and Stevens–Johnson syndrome (SJS) and toxic epidermal necrolysis (TEN) are considered the most severe delayed-type HSRs [10]. Dhopeshwarkar N et al. (2019) conducted an analysis of electronic health records and revealed that 1.1% of patients report drug-induced anaphylaxis, and ABs, nonsteroidal anti-inflammatory drugs (NSAIDs), and opiates were the main causative pharmacological groups [11]. A retrospective analysis based on the National Adverse Drug Reaction Monitoring System in China found the incidence of anaphylaxis to be 0.03%, and ABs, antineoplastic drugs, and contrast media were the most prevalent allergens [12]. ABs are also the most common triggers of delayed HSRs (SJS and TEN) [13,14]. 

The aim of our study was to estimate the proportion of spontaneous reports (SRs) with data on drug-induced anaphylaxis (SRsAs) among SRs of drug-induced allergy (SRsDIAs), the prevalence of pediatric and elderly SRsAs, and fatal outcomes among SRsAs, to discern drug categories associated with anaphylactic reactions and to assess patients’ characteristics (sex, age) using Russian National Pharmacovigilance database–Automatized Information System “Pharmacovigilance” (AIS). The AIS database accumulates reports of adverse drug reactions including SRsDIAs and SRsAs, reflecting real clinical practice tendencies. Analysis of the AIS database may contribute to a better understanding of the safety concerns of drug classes widely used in the population and may help to enhance the knowledge of healthcare professionals regarding the problem of drug-induced anaphylaxis.

## 2. Results

### 2.1. Analysis of SRsAs

A total of 27,727 SRsDIAs were detected in the electronic database during the study period (2 April 2019–21 June 2023). Of these, SRsAs accounted for 8.3% (*n* = 2304). The mean age of patients (SRsAs) was 48.2 ± 15.8 years (min —1 day, max—89 years), and 53.2% (*n* = 1226) were females.

An analysis of outcomes among SRsAs revealed recovery in 90.54% (*n* = 2086) and death in 9.46% (*n* = 218). 

An SRsA assessment revealed that seven main pharmacological groups were involved in anaphylaxis: antibacterials (ABs) for systemic use (44.6%, *n* = 1028), local anesthetics (LAs) (20%, *n* = 460), cyclooxygenase (COX) inhibitors (10.0%, *n* = 232), iodine-containing contrast media (ICCM) (6.6%, *n* = 153), cardiovascular (CV) drugs (6.2%, *n* = 143), central nervous system (CNS)-active drugs (1.5%, *n* = 35), and neuromuscular blocking agents (NMBAs) (1.4%, *n* = 33). Other drugs accounted for 9.5% (*n* = 220).

#### 2.1.1. Analysis of ABs Involved in Anaphylaxis

The mean age of patients with anaphylaxis due to ABs was 55.0 ± 14.9 years (min—1 day, max—89 years), and females accounted for 63.8% of cases (*n* = 655). The number of SRsAs where ABs were causative agents was 1028 (44.6%), and beta-lactam antibiotics were the most prevalent group (87.74%, *n* = 902). Ceftriaxone was the most common among all ABs (63.3%, *n* = 687). The structure of the ABs involved in anaphylaxis is shown in Table 1. Lethal outcomes were reported in 109 cases of SRsAs caused by ABs (10.6%).

#### 2.1.2. Analysis of LAs Involved in Anaphylaxis

LAs were identified as the main cause of anaphylaxis in 20% of SRsAs (*n* = 460). The mean age of patients with anaphylaxis due to LAs was 44.3 ± 14.9 years (min—5 months, max—84 years). Females accounted for 53.7% (*n* = 247). The most implicated LA by far was lidocaine (63.7%, *n* = 293). The structure of the LAs involved in anaphylaxis is shown in Table 2. Among the SRsAs caused by LAs, a lethal outcome was reported in 29 cases (6.3%).

#### 2.1.3. Analysis of COX Inhibitors Involved in Anaphylaxis

The mean age of patients listed in SRsAs with COX inhibitors as the main cause was 44.9 ± 14.5 (min—10, max—81) years, and 62.1% (*n* = 144) were females.

The number of SRsAs with COX inhibitors as the causative agent was 232, accounting for 10.07% of SRsAs. Among possible COX inhibitors, we considered NSAIDs, acetaminophen, and metamizole. Acetaminophen inhibits COX in the brain structures with analgesic and antipyretic effects [15]. Acetaminophen was the cause of SRsAs in the vast majority of cases (49, 21.1%). Similar results were seen for metamizole, another COX inhibitor without anti-inflammatory activity but with potent analgesic action [16]. It was detected in 48 cases (20.69%). Detailed information about the COX inhibitors involved in anaphylaxis is presented in Table 3. Among SRsAs where COX inhibitors were the causative drugs, a lethal outcome was reported in four cases (1.72%).

#### 2.1.4. Analysis of ICCM Involved in Anaphylaxis

ICCM were causative agents in 6.6% of SRsAs (*n* = 153). The mean age of patients was 55.4 ± 12.0 years (min—8.5 months, max—86 years), and 47.1% (*n* = 72) were females. The structure of the ICCM involved is presented in Table 4. A lethal outcome was reported in eight cases (5.2%).

#### 2.1.5. Analysis of CV Drugs Involved in Anaphylaxis

SRsAs with CV drugs being causative agents accounted for 6.2% (*n* = 143) of SRsAs. The mean age of patients was 57.7 ± 16.2 (min—19, max—86), and females accounted for 76.9% of cases (*n* = 110). The highest frequency was reported for angiotensin-converting enzyme inhibitors, ACEIs (20.3%, *n* = 29), followed by beta-blockers (15.4%, *n* = 22) and calcium channel blockers (14.7%, *n* = 21). Table 5 contains data on the CV drugs involved in anaphylaxis. Among SRsAs due to CV drugs, a lethal outcome was detected in 13 cases (9.1%).

#### 2.1.6. Analysis of CNS-Active Drugs Involved in Anaphylaxis

SRsAs analysis revealed CNS-active drugs being causative agents in 35 cases (1.5%). The mean age of patients was 54.6 ± 17.8 years (min—23, max—82). The structure of the drugs involved in anaphylaxis is indicated in Table 6. Among the CNS-active drugs, the highest prevalence was reported for the synthetic opioid, fentanyl (57.1%, *n* = 20). A lethal outcome was reported in five cases (14.3%).

#### 2.1.7. Analysis of NMBAs Involved in Anaphylaxis

NMBAs were detected as causative drugs in 1.2% of SRsAs (*n* = 28). The mean age of patients was 47.4 ± 14.3 years (min—22, max—71). The highest prevalence was reported for rocuronium (*n* = 10)—30.3%. The NMBAs involved in anaphylaxis are presented in Table 7. A lethal outcome was detected in 28.6% (*n* = 8) of cases.

### 2.2. Analysis of Fatal SRsAs

The total number of fatal SRsAs was 218 (9.5%). Reports analysis revealed 56.4% of fatal SrsAs were of women (*n* = 123), and the mean age was 48.0 ± 16.7 (min—1 month, max—86 years). To estimate the effect of gender as a variable, Pearson’s chi-squared test of independence was used, and no statistically significant difference (*p* = 0.318) was revealed. 

Two cases of death were observed in pregnant women (0.9%), 17 cases in children (7.8%), and 30 (13.8%) in the elderly. The leading causative groups were ABs (50%, *n* = 109), LAs (13.3%, *n* = 29), and CV drugs (6.0%, *n* = 13). The drugs detected in fatal SRsAs are shown in Table 8.

The mean age of patients in fatal SrsAs for each causative group of drugs is listed in Table 9 together with sex distribution (%). The youngest age was reported in fatal SRsAs where LAs were causative drugs and the oldest where CV drugs were the cause. Females predominated in all groups except fatal SRsAs with CV drugs, where 69.2% (*n* = 9) were males.

**Figure 1:**
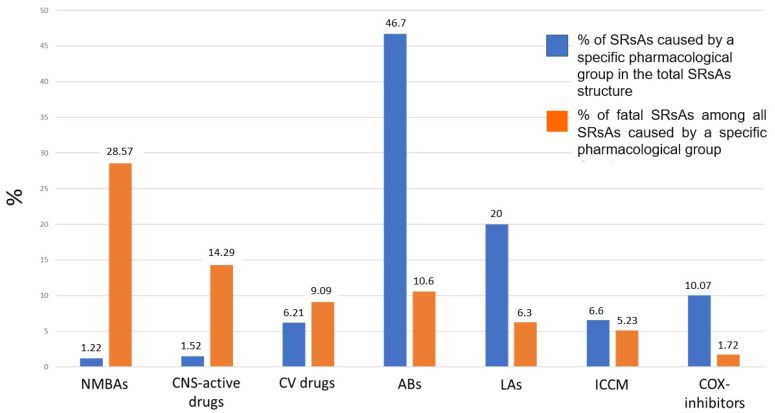
Comparison of % of spontaneous reports (SRs) with data on drug-induced anaphylaxis (SRsAs) due to a specific pharmacological group among the total SRsAs structure and % of fatal SRsAs among all SRsAs caused by a specific pharmacological group.

The highest proportion of fatal SRsAs was revealed in SrsAs where NMBAs and CNS-active drugs were the causative agents, notwithstanding the fact that SRsAs caused by these pharmacological groups accounted for only 1.2 and 1.5% in the total structure, respectively (Figure 1).

### 2.3. Analysis of Pediatric SRsAs

There were 3443 pediatric SRsDIAs (reports of patients ≤ 18 years) identified in the AIS database, and SRsAs accounted for 3.9% of these (*n* = 133). Among all SrsAs, pediatric SRsAs accounted for 5.8%. 

The mean age was 11.8 ± 4.5 years (min—1 day, max—18 years), and females accounted for 51.9% (*n* = 69). An outcome analysis revealed recovery in 87.2% (*n* = 116) and death in 12.8% (*n* = 17) of cases. 

The most prevalent groups of drugs involved in anaphylaxis in children were ABs (42.9%, *n* = 57), LAs (12.8%, *n* = 17), and COX inhibitors (6.0%, *n* = 8). Other drugs accounted for 38.3 (*n* = 51).

ABs were the leading causative pharmacological group in children and the absolute majority of reactions (87.7%) were due to ABs (Table 6). The mean age of children with anaphylaxis due to ABs was 16.0 ± 12.6 years (min—1 day, max—18 years), females accounted for 51.2% (*n* = 29) of cases. The structure of ABs involved in anaphylaxis in children is given in Table 10. 

A lethal outcome due to ABs was reported in eight cases (14.0%, 8/57), the mean age was 7.4 ± 5.4 (min—15 months, max—16 years), and males accounted for 85.7% (*n* = 6) of cases. Ceftriaxone was detected in seven cases and amoxicillin clavulanate in 1.

Among LAs, the leading agent was lidocaine, responsible for 52.9% of cases (Table 11). The mean age of children in SRsAs with LAs was 9.9 ± 4.6 (min—5 months, max—17 years), and males accounted for 64.7% (*n* = 11) of cases. Fatal SRsAs included only lidocaine (3/17, 17.6%), the mean age was 10.0 ± 3.3 (min—5, max—13) years, and males accounted for 66.7% (*n* = 2) of cases.

There were only eight SRsAs with COX inhibitors in the pediatric population (acetaminophen–50% (*n* = 4), ketorolac—25% (*n* = 2), and metamizole–25% (*n* = 2)). The mean age was 15.0 ± 2.0 (min—10, max—18) years, and females accounted for 62.5% (*n* = 5). There were no lethal outcomes among SRsAs with COX inhibitors as causative agents in children.

### 2.4. Analysis of SRsAs in the Elderly

There were 3307 SRsDIAs of patients ≥65 years detected in the AIS database and SRsAs accounted for 2% of these (*n* = 65). Among all SRsAs, SRsAs of the elderly accounted for 2.8%. The mean age was 73.0 ± 5.3 (min—65, max—89) years, and females accounted for 43.5% (*n* = 27) of cases. An outcomes analysis revealed recovery in 53.8% (*n* = 35) and death in 46.2% (*n* = 30) of cases. 

The most common causative pharmacological groups were ABs (40%, *n* = 26), CV drugs (20%, *n* = 13), and ICCM (12.3%, *n* = 8). The drugs involved in anaphylaxis in the elderly are demonstrated in Table 12. 

Among the records with lethal outcomes in the elderly, 53.3% (16/30) were reported in females. The majority of fatal SRsAs revealed ABs as causative agents (the only drug was ceftriaxone, 31.4%, *n* = 16). ICCM were reported in 15.7% (*n* = 8), pentoxifylline in 3.9% (*n* = 2), amiodarone in 2.0% (*n* = 1), bupivacaine in 2.0% (*n* = 1), cisplatin in 1.5% (*n* = 1), and oxaliplatin in 1.5% (*n* = 1).

## 3. Discussion

Our results revealed anaphylaxis in 8.3% of SRsDIAs registered in the AIS “Pharmacovigilance” database (2304/27,727) during the study period, 2 April 2019–21 June 2023. Fatal SRsAs were reported in 9.5% (*n* = 218) of cases. The analysis of SRsAs across 2304 records revealed similar age and sex characteristics between those who survived and those who died (48.2 ± 15.8 years, 53.2% females vs. 48.0 ± 16.7 years, 56.4% females). 

Based on the results of an 8-year post hoc analysis of the MEREAFaPS Study (“Monitoraggio Epidemiologico delle Reazioni e degli Eventi Avversi da Farmaci in Pronto Soccorso”—“Epidemiological Monitoring of Adverse Drug Reactions and Events leading to Emergency Department”, a multicentre study of active pharmacovigilance, Italy) database (2012–2019), the mean age of population with anaphylaxis was 55.7 ± 17.7 years, and females accounted for 52.4% of cases [17]. The mean age of patients with drug-induced anaphylaxis in China was determined as 47.6 years (Beijing Pharmacovigilance Database analysis), and 52.7% were females [18]. An analysis of records with drug-induced anaphylaxis in West Pomerania, Poland, revealed the mean age of the affected population to be 40.5 years, and 54.4% were females [19]. Female dominance in the structure of patients with drug-induced anaphylaxis (57.9%) was also proved by the results of an analysis of electronic health records (EHRs) of a large United States healthcare system [11] and by the results of a Tunisian retrospective study (males/female ratio was 0.6)). In the latter study, the patients were younger than in most other published works—the mean age was 33.52 years [20]. An analysis of drug-induced anaphylaxis in a Vietnamese Pharmacovigilance Database revealed a prevalence of 51.8% in patients between 20 and 60 years old, and 53.2% were female [21]. An analysis of the Korean Health Insurance Review and Assessment Service (HIRA) (January 2011 to December 2019) indicated that the mean age of patients with drug-induced anaphylaxis was 52 years, and 55.2% were female [22]. 

ABs are among the most common triggers of drug-induced anaphylaxis. Their leading role was replicated in both retrospective and prospective studies [8,23,24,25]. Our results revealed ABs to be the main cause of anaphylaxis in all age groups (44.6% (*n* = 1028) in total SRsAs, 42.9% (*n* = 57) in pediatric SRsAs, and 40.0% (*n* = 26) in the elderly), and among fatal SRsAs (50% (*n* = 109)). According to our study, the most common agent among ABs causing anaphylaxis was ceftriaxone. 

Zhao et al. (2018) revealed ABs to be the main group of drugs involved in anaphylaxis (39.3%), followed by traditional Chinese medicines (11.9%), radiocontrast agents (11.9%), and antineoplastic agents (10.3%) [18]. Among all the drugs investigated, cephalosporins were the leading agents (34.5%) [18].

Based on an FDA Adverse Event Reporting System (FAERS) analysis, drug-induced anaphylaxis was reported in 0.27% of all adverse drug events (47,496/17,506,002), and causative drugs included ABs (14.87%)), monoclonal antibodies (13.06%), and COX inhibitors (NSAIDs and acetaminophen—8.83%). Anaphylaxis deaths were associated with ABs, radiocontrast agents, and intraoperative agents, and the rate of fatal cases was 6.28% (2984/47,496) [23]. 

Pagani et al. (2022) defined the leading role of ABs in ARs development (53.78%), and penicillins were the most prevalent (66.67%), followed by cephalosporins (21.10%) and fluoroquinolones (8.56%) [17]. Penicillins were the cause of ARs in 50% of cases according to the data of Wong et al. (2019), and sulfonamides and cephalosporines were other common causes [9]. 

Cephalosporins are associated with the full spectrum of HSRs and their immunogenicity is mainly defined by the presence of R1 side chains in the structure of the molecule [26]. Cephalosporins have been proven to be common triggers of ARs in adults and children [26,27,28,29], and third-generation agents including ceftriaxone were among the main inducers of allergic reactions reported in hospitals in South Korea [30]. An analysis of the Korean Adverse Event Reporting System (KAERS) and HIRA database revealed incidence rates for hypersensitivity reactions including anaphylaxis to cefaclor, other second-generation cephalosporins, and third-generation cephalosporins to be 1.17/10,000 persons (0.38/10,000 persons), 3.57/10,000 persons (0.38/10,000 persons), and 5.82/10,000 persons (0.61/10,000 persons), respectively [31]. Another Korean study revealed five common medication risk factors for drug-induced anaphylaxis including cephalosporine cefaclor, ICCM (iopromide, iohexol, iomeprol), and tolperisone [22]. Cephalosporines were defined as the most common ABs causing ARs based on the results of a China Hospital Pharmacovigilance System analysis [12]. Third-generation cephalosporines were determined as the main cause of drug-induced anaphylaxis based on the results of a Vietnamese Pharmacovigilance Database [21]. 

In our study, LAs were reported in 20.0% of SRsAs (*n* = 460). They ranked second in the list of causative agents among total SRsAs, pediatric SRsAs, and fatal SRsAs. LAs are typical triggers of anaphylaxis in dentistry practice [32], though the risk of true IgE-mediated allergy was shown to be lower than 1% [33,34,35,36,37]. Amide local anesthetics are less involved in hypersensitivity reactions compared with ether local anesthetics, and among the amides, lidocaine is known to be the most associated with severe allergic reactions. Other amides are safer in this respect, though published data demonstrates that some cause anaphylactic shock, which was demonstrated for mepivacaine [38] and bupivacaine [39]. A French Pharmacovigilance Database System analysis revealed a twentyfold growth in the number of reports on anaphylactic reactions involving local anesthetics from 1985 to 2020, and lidocaine was reported to be the most common cause (81.49%) [40]. The leading role of lidocaine in severe HSRs and anaphylaxis development in dental practice was stated in the work by Matveev et al. (2020) [41]. Anesthetics (first, lidocaine, second, bupivacaine) were reported to be among the top eight pharmacotherapeutic groups involved in anaphylaxis based on the results of a Vietnamese Pharmacovigilance Database analysis [21]. 

NSAIDs are a common cause of hypersensitivity [25], responsible for a considerable proportion of anaphylaxis in clinical practice of tertiary care hospitals and emergency departments [8,21,24,42]. Mechanisms of NSAID-induced anaphylaxis are unknown in most cases, though accelerated IgE formation is supposed for aril-propionic agents (ibuprofen, ketoprofen, flurbiprofen, naproxen, fenoprofen, oxaprozin, aceclofenac, and fenclofenac) and transient competitive inhibition of prostanoid biosynthesis may also contribute to symptoms [42]. Another hypothesis suggests that NSAIDs may increase adenosine levels activating adenosine receptors, which may lead to degranulation of mast cells [43].

Our data revealed COX inhibitors (NSAIDs, acetaminophen, and metamizole) to be the third most prevalent causative group among total (10.1%, main drug: acetaminophen) and pediatric SRsAs (6.0%, main drug: acetaminophen), and the fourth among elderly SRsAs (7.7%, main drug: diclofenac). Fatal SRsAs analysis revealed COX inhibitors only in 1.8% (two cases: diclofenac, one case: ibuprofen). In the USA, NSAIDs (ibuprofen and naproxen) were the second most prevalent cause of anaphylactic reactions (13.0%) after ABs (61%) [11], while in Poland, NSAIDs were the main causative pharmacological group (acetylsalicylic acid, ketoprofen, metamizole, and ibuprofen) [19]. These results are supported by the FAERS database analysis (study period 1999 to 2019) by Yu RJ et al. (2021), which revealed acetaminophen and NSAIDs (acetylsalicylic acid, celecoxib, diclofenac) among the top 50 drugs causing anaphylaxis [23]. Based on the analysis of a Vietnamese Pharmacovigilance Database, NSAIDs were found to be the second most prevalent pharmacological group involved in drug-induced anaphylaxis [21]. Published data indicate that in children, NSAIDs are the second most significant pharmacological group causing anaphylaxis after ABs [44]. Our results revealed acetaminophen to be the leader among COX inhibitors in total SRsAs and pediatric SRsAs, but no fatal cases involving acetaminophen were detected. An analysis of the EudraVigilance Database (2007–2018 years) found that acetaminophen-induced anaphylaxis was most common in the age group 18–64 years, and among acetaminophen-induced Ars, anaphylaxis was the second most common cause of death after hepatic failure with shock [45]. A systematic review of 85 studies reporting hypersensitivity reactions to acetaminophen revealed that acetaminophen hypersensitivity reaction prevalence among children was 10.1% (95% confidence interval 4.5–15.5) [46]. A retrospective analysis of 159 validated spontaneous reports in children (database of the German Federal Institute for Drugs and Medical Devices) revealed another COX inhibitor, ibuprofen, to be the main drug responsible for anaphylaxis development [47]. 

According to our results, ICCM were identified in 6.6% of SRsAs (*n* = 153), with the most common agents being iopromide and iohexol. ICCM were the third most prevalent group of causative drugs in the elderly SRsAs (12.3%). This group of agents is known to mediate severe hypersensitivity reactions which may lead to a lethal outcome [48,49]. The ICCM group was reported to be a leader among drugs involved in anaphylaxis due to the results of a 13-year period analysis of the Japanese Adverse Drug Event Report (JADER) database [50]. Based on the results of a 10-year study in China, total radiocontrast agents accounted for 11.9% and ICCM for 9.5% [18]. Pagani et al. (2022) demonstrated an association of anaphylaxis with radiology contrast agents in 6.92% of cases [17]. Nguyen et al. (2019) reported contrast media to be in fourth place among all pharmacological groups causing drug-induced anaphylaxis [21]. A FAERS database analysis reported iohexol, iopamidol, and iopromide to be among the top 50 drugs involved in anaphylaxis [23]. Published studies suggest that anaphylaxis due to radiocontrast media is more common in older age and with repeated drug exposure [51]. The mechanisms of anaphylaxis development by ICCM are still unclear. Published data suggest the activation of mast cells and basophils, direct release of histamine, tryptase, and other allergy mediators, activation of the complement systems, and activation of the XII clotting system with subsequent bradykinin release, as well as the formation of pseudoantigens [52]. 

In our study, CV drugs were the fourth most significant group involved in anaphylaxis in total SRsAs (6.2%, *n* = 143) with the most common groups being ACEIs (enalapril, captopril, perindopril), beta-blockers (bisoprolol, metoprolol, atenolol), and calcium channel blockers (nifedipine, amlodipine, verapamil). In the elderly, CV drugs were the second most prevalent group causing anaphylaxis (20%, *n* = 13). ACEIs and beta-blockers are known causes of anaphylaxis in clinical practice [53]. A systematic review and meta-analysis of observational studies stated that beta-blockers and ACEIs increase the severity of anaphylaxis (beta-blockers, odds ratio [OR] 2.19, 95% confidence interval [CI] 1.25–3.84; ACEIs, OR 1.56, 95% CI 1.12–2.16) [54]. Anaphylaxis severity was shown to be increased with ACEI intake along with the presence of such factors as mastocytosis, and high fever prior to anaphylaxis [55]. Published studies based on pharmacovigilance databases show a lower significance for CV drugs in anaphylaxis compared with our results. A FAERS analysis reported no CV agents among the top 50 drugs causing anaphylaxis [23]. An analysis of the Vietnamese Pharmacovigilance Database revealed cardiac therapy agents to be in 22nd place among drugs involved in anaphylaxis [21]. A 10-year retrospective analysis of the Beijing Pharmacovigilance Database reported cardiovascular medications accounted for 0.9% of drug-induced anaphylaxis [18]. Possible mechanisms of CV-drug-induced anaphylaxis are multiple and depend on the pharmacological group. For example, statins reduce plasma levels of low-density lipoprotein which may lead to increased concentration of platelet-activating factor [43]. ACE inhibitors are known to accumulate bradykinin levels leading to angioedema development; however, considering anaphylaxis, it is thought that ACE inhibitors, as well as beta-blockers, may act through activation of the high-affinity IgE receptor FcεRI [43]. 

In our study, the total number of SRsAs with CNS-active drugs as causative agents was 35 (1.5%) with the leading roles being played by fentanyl, diazepam, and tramadol. Published clinical studies reveal a relatively low incidence of fentanyl-associated anaphylaxis [56,57], mainly single cases are reported [56,58,59,60]. A low frequency of anaphylaxis is also known for benzodiazepines [61], though diazepam is considered to be a more common cause of allergy compared with midazolam [62]. Pagani et al. (2022) indicated a frequency of anaphylaxis due to tramadol equal to 0.32%, 2/608 [17], and a literature analysis revealed only a few cases of tramadol-induced anaphylaxis [63,64,65]. Conversely, some studies based on analyses of pharmacovigilance databases reveal a significant role for several drugs affecting CNS. A FAERS database analysis reported fentanyl, midazolam, propofol, and sufentanyl among the top 50 drugs involved in anaphylaxis [23]. An analysis of the Vietnamese Pharmacovigilance Database indicated analgesic opioids and psychostimulants to be in the 18th and 19th positions among groups involved in anaphylaxis [21]. Opioids may lead to direct histamine release, though for the high-potency opiate fentanyl, or benzodiazepines, this is not common. Possible mechanisms of CNS-active drug-induced anaphylaxis are still under debate [43,66].

In our study, NMBAs accounted for 1.43% of cases (*n* = 33), and the most prevalent were rocuronium and suxamethonium. NMBAs are the most frequent allergens responsible for acute hypersensitivity reactions during anesthesia [67,68,69] and leading causative agents for perioperative anaphylaxis [70]. A French pharmacovigilance survey from 2000 to 2012 revealed suxamethonium and rocuronium to be the most common NMBAs causing ARs [71]. Atracurium, rocuronium, and succinylcholine were listed among the top 50 drugs involved in anaphylaxis based on a FAERS analysis [23]. Published data revealed that NMBA-induced anaphylaxis may be mediated by IgG-dependent neutrophil activation with or independently of IgE-dependent mast cell/basophil activation [4]. 

According to our analysis of fatal SrsAs, most cases were due to ABs (and beta-lactams among ABs), which is in complete accordance with published data. Beta-lactam antibiotics, muscle relaxants, and injected radiocontrast media were the main triggers of fatal drug anaphylaxis based on an analysis carried out by Turner et al. (2017) [72], and the higher prevalence of ABs among drugs involved in total ARs and fatal ARs is also proved by the vast majority of reported studies in adults and children [11,17,18,23,44,73,74].

Published data indicate that drug-induced anaphylaxis is associated with more lethal cases than food-induced and venom-induced anaphylaxis [75]. The actuality of the problem is supported by the increasing number of fatal cases reported in modern studies. A systematic review of 46 observational studies reported an increased frequency of deaths due to drug-induced anaphylaxis during the study period (IRR per year, 1.02; 95% CI, 1.00–1.04), and the highest rates were detected for the Australian region [76]. Jerschow et al. (2014) stated a significant increase in cases of fatal drug-induced anaphylaxis over a twelve-year period (from 0.27 (95% CI, 0.23–0.30) per million in 1999 to 2001 to 0.51 (95% CI, 0.47–0.56) per million in 2008 to 2010 (*p* < 0.001)) [77]. The percentage of fatal SrsAs reported in our study (9.5%) exceeds published values. A FAERS database analysis revealed 6.28% (2984/47,496) reports of anaphylaxis followed by death [23], a Brazilian Hospital Information System analysis, 5.8% [78], a Beijing Pharmacovigilance Database analysis, 3.3% (39/1189) [18], and a Vietnamese Pharmacovigilance Database analysis, about 2.3% (111/4873) [21]. The rate of fatal drug-induced anaphylaxis in Spain was 1.02% [79], and an analysis of the Latin American anaphylaxis registry revealed that only 0.3% of cases were fatal [80]. 

Our study has some limitations. First, the retrospective design of the study based on the analysis of SRsAs entered in the AIS “Pharmacovigilance” database made it impossible to evaluate in all cases the effect of concomitant medications and comorbidities, laboratory tests performed, and to estimate risks of anaphylaxis in different populations. We should also state that it is sometimes difficult to distinguish the cause of an adverse reaction, an anaphylactic reaction or a direct vascular reaction leading to a profound hypotension. 

It is worth noting that the number of SRsAs reported in our study was based on the analysis of spontaneous reporting records and thus cannot completely reflect the prevalence of anaphylaxis in real clinical practice. Reported proportions of drugs involved in anaphylaxis in our study may be determined not by their true safety profile, but by the frequency of their prescribing. For example, the leading causative agent determined in our study was ceftriaxone; however, it is also one of the most prescribed drugs to treat various infectious diseases worldwide, ranging from 2.5% of therapeutic prescriptions in Northern Europe to 24.8% in Eastern Europe. It is also the most prescribed AB for surgical prophylaxis (34.4% of AB prescriptions in Eastern Europe, 24.8% in Southern Europe, 23.6% in West and Central Asia, and 19.7% in Northern Africa) [81]. A promising approach to assess the real-world prevalence of ARs due to a drug may be based on a parallel assessment of drug consumption rates.

## 4. Materials and Methods

### 4.1. Data Source

The Federal Service for Surveillance in Healthcare (Roszdravnadzor) is responsible for drug safety and effectiveness monitoring in the Russian Federation. Spontaneous/voluntary adverse reaction (AR) reporting is regulated by legislation in the Russian Federation. All reports are directed to the AIS “Pharmacovigilance” database, which is a national pharmacovigilance database that was established in 2008, and its structure, functioning, and management comply with ICH E2B (R3) standard [82]. The AIS “Pharmacovigilance” database uses MedDRA version 25.0 as a reference tool [83]. Drugs are identified by brand names and international nonproprietary names (INN), which are both selected automatically by reporters when they fill out the official reporting form. Drug categories are determined in accordance with ATC classification. Causality assessment is made in the AIS “Pharmacovigilance” database by the built-in WHO algorithm and Naranjo algorithm. Signal detection is performed using built-in quantitative statistical methods (proportional reporting ratio, PRR; reporting odds ratio, ROR; reduced rank regression, RRR). The AIS “Pharmacovigilance” database receives ARs reports on all drugs registered and approved for use in the Russian Federation, and cases that occurred abroad. SRs may come from medical professionals, pharmaceutical companies, patients, or their representatives. However, in June 2023 the total number of individual case safety reports in AIS was >2,100,000, and no cases of reports made by patients or their representatives were presented. Most reports were generated by healthcare workers (mainly from hospitals).

### 4.2. Definitions

For this study, we used the following definitions [84]:

1. “Adverse reaction—A response to a medicinal product, which is noxious and unintended. Adverse reaction may arise from use of the product within or outside the terms of the marketing authorization or from occupational exposure. Use outside the marketing authorization includes off-label use, overdose, misuse, abuse and medication errors.”
2. “Causality: In accordance with ICH-E2A, the definition of an adverse reaction implies at least a reasonable possibility of a causal relationship between a suspected medicinal product and an adverse event. An adverse reaction, in contrast to an adverse event, is characterized by the fact that a causal relationship between a medicinal product and an occurrence is suspected. For regulatory reporting purposes, as detailed in ICH-E2D, if an event is spontaneously reported, even if the relationship is unknown or unstated, it meets the definition of an adverse reaction. Therefore, all spontaneous reports notified by healthcare professionals or consumers are considered suspected adverse reactions, since they convey the suspicions of the primary sources, unless the reporters specifically state that they believe the events to be unrelated or that a causal relationship can be excluded.”
3. “A spontaneous report is an unsolicited communication by a healthcare professional, or consumer to a competent authority, marketing authorisation holder or other organization (e.g., regional pharmacovigilance center, poison control center) that describes one or more suspected adverse reactions in a patient who was given one or more medicinal products. It does not derive from a study or any organized data collection systems.”

### 4.3. Study Design and Data Selection

Study design: a retrospective, descriptive study of SRs accumulated in the AIS “Pharmacovigilance” database. Study period: 2 April 2019–21 June 2023. 

**Figure 2:**
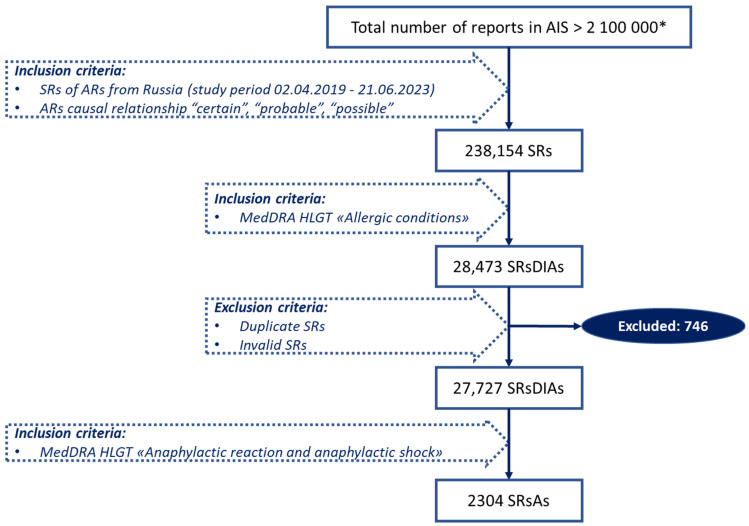
Flowchart of SRsAs selection from AIS “Pharmacovigilance”. * Number given for the study period 2 April 2019–21 June 2023.

Data selection was made using the steps described in Figure 2. First, from the total number of reports in the AIS “Pharmacovigilance” database, SRs from the Russian Federation only were extracted with the obligative inclusion criterion of a high probability of a causal relationship (“certain”, “probable”, “possible”). Probability assessment was performed automatically in the AIS “Pharmacovigilance” database using the built-in Naranjo algorithm. Second, using the MedDRA high-level group term (HLGT) “Allergic conditions”, we defined the total number of SRs describing drug-induced allergy (SRsDIAs). Then, we excluded duplicate and invalid reports to obtain the exact number of SRsDIAs that occurred in the Russian Federation. Validity was determined according to paragraph VI.B.2 of the EMA “Guideline on good pharmacovigilance practices” and paragraph 407 of the Eurasian Economic Union “Good pharmacovigilance practice”, which state that information in an SR must contain at least 4 elements: identifiable reporter; identifiable patient; at least one suspected drug; an at least one suspected ADR. If any of these 4 elements is absent, the report is considered invalid [84,85]. In the next step, we used the MedDRA HLGT “Anaphylactic reaction and anaphylactic shock” to detect SRs with anaphylaxis (SRsAs). 

The diagnosis of anaphylaxis in the reports directed to the Russian Pharmacovigilance database is established by healthcare professionals/physicians in accordance with the clinical criteria for the diagnosis of anaphylaxis accepted in the Russian Federation. These criteria are fully consistent with the criteria developed by the World Allergy Organization Anaphylaxis Guidance 2020 [1]. For all patients with anaphylaxis, physicians are responsible for performing an immunological study and its results are required to be included in the corresponding section of SRsAs. In cases of patients who died from anaphylaxis, healthcare professionals/physicians responsible for the SRsA filing should include autopsy data in the corresponding section of the SRsAs. Based on this information, our study is relevant to the disclosure of anaphylaxis data in Russia. 

### 4.4. Drug Identification and Analyzed Categories

For this study, we used INNs of suspected drugs, and groups were distinguished according to the ATC classification. Drug identification did not distinguish between dosage forms or routes of administration.

Patient demographic information and data on causative drugs were extracted from the sample including SrsAs. All identified SrsAs were first analyzed. Then, two age categories were defined: elderly (SRsAs describing patients ≥ 65 years) and pediatric (SRsAs describing patients ≤ 18 years). The reported age was described in days, weeks, and years. Fatal SRsAs were also estimated (SRsAs with a lethal outcome due to anaphylaxis).

### 4.5. Statistical Analysis

Descriptive statistics was used for all analyzed parameters; qualitative variables were described using absolute (n) and relative (%) values. All statistical analyses were performed using Microsoft Excel 2019. The percentage of SRsAs among SRsDIAs was estimated, mean age and sex differences were analyzed, and the structure of the causative drugs was detected. Pearson’s chi-squared test was used to assess gender as a variable in patients with fatal SRsAs.

## 5. Conclusions

The SR analysis performed in our work revealed anaphylaxis to account for 8.3% of all drug-induced allergic reactions. In terms of structure, the leading drugs involved were ABs, LAs, ICCM, COX inhibitors, CV drugs, and CNS-active drugs. The number of pediatric SRsAs was almost twice that of SRsAs in the elderly (5.8% vs. 2.8%). Our results proved a higher prevalence of females with drug-induced anaphylaxis in all analyzed categories of SRsAs except the elderly (43.5%). Fatal SRsAs were reported in 9.5% of cases and were mainly caused by ABs, LAs, and CV drugs. The highest percentage of deaths was observed in the elderly (46.2% (*n* = 30)), while in children, it was 3.6 times lower (12.78% (*n* = 17)). National pharmacovigilance databases and EHRs are important tools to assess the structure of drugs involved in various allergic reactions including HSRs and to obtain information on the demographic characteristics of patients [86], although more objective results may be obtained by taking into account data on the actual consumption of the relevant drugs in real clinical practice.

## Figures and Tables

**Table 1:** ABs involved in anaphylaxis.

ABs	*n* (Total—1028)	%
*Beta-lactams*	*902*	*87.7*
Ceftriaxone	687	66.8
Cefotaxime	87	8.5
Cefazolin	39	3.8
Ampicillin sulbactam	17	1.7
Cefepime	15	1.5
Cefuroxime	10	1.0
Meropenem	10	1.0
Amoxicillin clavulanate	8	0.8
Cefoperazone sulbactam	7	0.7
Ertapenem	5	0.5
Cefoperazone	4	0.4
Ampicillin	4	0.4
Cefepime sulbactam	3	0.3
Amoxicillin sulbactam	2	0.2
Piperacillin tazobactam	2	0.2
Cephalexin	1	0.1
Cefixime	1	0.1
*Other*	*126*	*12.3*
Vancomycin	28	2.7
Ciprofloxacin	22	2.1
Levofloxacin	18	1.8
Metronidazole	14	1.4
Linezolid	7	0.7
Amikacin	7	0.7
Nitrofurantoin	7	0.7
Fosfomycin	5	0.5
Sulfamethoxazole trimethoprim	3	0.3
Tigecycline	3	0.3
Polymyxin B	2	0.2
Kanamycin	2	0.2
Amphotericin B	2	0.2
Gentamicin	1	0.1
Ofloxacin	1	0.1
Erythromycin	1	0.1
Clindamycin	1	0.1
Rifampicin	1	0.1
Isoniazid	1	0.1

ABs—antibacterials; *n*—number.

**Table 2:** LAs involved in anaphylaxis.

LAs	*n* (Total—460)	%
Lidocaine	293	63.7
Procaine	68	14.8
Articaine	61	13.3
Ropivacaine	16	3.5
Bupivacaine	14	3.0
Mepivacaine	8	1.7

LAs—local anesthetics; *n*—number.

**Table 3:** COX inhibitors involved in anaphylaxis.

COX-Inhibitors	*n* (Total—232)	%
Acetaminophen	49	21.1
Metamizole	48	20.7
Ibuprofen	35	15.1
Diclofenac	29	12.5
Ketorolac	26	11.2
Acetylsalicylic acid *	25	10.8
Ketoprofen	6	3.0
Celecoxib	5	2.2
Aceclofenac	3	1.3
Meloxicam	3	1.3
Lornoxicam	2	0.9
Nimesulide	1	0.4

* All cases included the use of acetylsalicylic acid in the dose of 500 mg as an antipyretic and analgesic agent only. COX—cyclooxygenase; *n*—number.

**Table 4:** ICCM involved in anaphylaxis.

ICCM	*n* (Total—153)	%
Iopromide	97	63.4
Iohexol	39	25.5
Iomeprol	13	8.5
Iodixanol	2	1.3
Ioversol	1	0.7
Iopamidol	1	0.7

ICCM—iodine-containing contrast media; *n*—number.

**Table 5:** CV Drugs involved in Anaphylaxis.

CV Drug	*n* (Total—143)	%
*ACEIs*	*29*	*20.3*
Enalapril	17	11.9
Captopril	11	7.8
Perindopril	1	0.7
*Beta-blockers*	*22*	*15.4*
Bisoprolol	13	9.1
Metoprolol	5	3.5
Atenolol	3	2.1
Propranolol	1	0.7
*Calcium Channel Blockers*	*21*	*14.7*
Nifedipine	9	6.3
Amlodipine	8	5.6
Verapamil	4	2.8
*Potassium-magnesium-asparaginate*	*19*	*13.3*
*Antiarrhythmics*	*12*	*8.4*
Amiodarone	10	7.0
Digoxin	1	0.7
Propafenone	1	0.7
*Diuretics*	*11*	*7.7*
Furosemide	7	4.9
Spironolactone	3	2.1
Hydrochlorothiazide	1	0.7
*Sartans (Losartan)*	*8*	*5.6*
*Statins*	*6*	*4.2*
Rosuvastatin	4	2.8
Atorvastatin	2	1.4
*Alpha-2 adrenergic receptor agonist (Clonidine)*	*4*	*2.8*
*Indirect oral anticoagulant (Warfarin)*	*4*	*2.8*
*Direct oral anticoagulants*	*2*	*1.4*
Rivaroxaban	1	0.7
Apixaban	1	0.7
*Unfractionated heparin*	*2*	*1.4*
*Antiplatelet drugs*	*2*	*1.4*
Clopidogrel	1	0.7
Ticagrelor	1	0.7
*Thrombolytic agent (Alteplase)*	*1*	*0.7*

CV—cardiovascular; *n*—number.

**Table 6:** CNS-active Drugs involved in Anaphylaxis.

CNS-Active Drugs	*n* (Total—35)	%
Fentanyl	20	57.1
Diazepam	4	11.4
Tramadol	4	11.4
Midazolam	3	8.6
Venlafaxine	1	2.9
Droperidol	1	2.9
Carbamazepine	1	2.9
Levetiracetam	1	2.9

CNS—central nervous system; *n*—number.

**Table 7:** NMBAs involved in Anaphylaxis.

NMBAs	*n* (Total—28)	%
Rocuronium	10	35.7
Atracurium	8	28.6
Suxamethonium	5	17.9
Cisatracurium	5	17.9

NMBAs—neuromuscular blocking agents; *n*—number.

**Table 8:** Drugs involved in Fatal SRsAs.

Drug	*n* (Total—218)	%
*ABs*	*109*	*50.00*
Ceftriaxone	68	31.2
Cefotaxime	13	6.0
Fosfomycin	5	2.3
Amoxicillin clavulanate	3	1.4
Levofloxacin	3	1.4
Ciprofloxacin	3	1.4
Cefazolin	3	1.4
Ampicillin sulbactam	2	0.9
Ertapenem	2	0.9
Amphotericin B	1	0.5
Meropenem	1	0.5
Tigecycline	1	0.5
Sulfamethoxazole trimethoprim	1	0.5
Vancomycin	1	0.5
Metronidazole	1	0.5
*LAs*	*29*	*13.3*
Lidocaine	19	8.7
Bupivacaine	7	3.2
Articaine	1	0.5
Procaine	1	0.5
Ropivacaine	1	0.5
*CV drugs*	*13*	*6.0*
Beta-blockers	3	1.4
Bisoprolol	2	0.9
Metoprolol	1	0.5
ACEi (Enalapril)	3	1.4
Sartans (Losartan)	1	0.5
Calcium Channel Blockers (Amlodipine)	2	0.9
Antiarrhythmics (Amiodarone)	1	0.5
Statins (Rosuvastatin)	1	0.5
Antiplatelet drugs (Ticagrelor)	1	0.5
Alpha-2 adrenergic receptor agonist (Clonidine)	1	0.5
*NMBAs*	*8*	*3.7*
Rocuronium	4	1.8
Suxamethonium	2	0.9
Atracurium	1	0.5
Cisatracurium	1	0.5
*ICCM*	*8*	*3.7*
Iopromide	4	1.8
Iohexol	3	1.4
Iomeprol	1	0.5
*CNS-active drugs*	*5*	*2.3*
Fentanyl	2	0.9
Diazepam	1	0.5
Levetiracetam	1	0.5
Midazolam	1	0.5
*COX inhibitors*	*4*	*1.8*
Diclofenac	2	0.9
Ibuprofen	1	0.5
Acetylsalicylic acid	1	0.5
*Other drugs*	*42*	*19.3*

ABs—antibacterials; LAs—local anesthetics; CV—cardiovascular; NMBAs—neuromuscular blocking agents; ICCM—iodine-containing contrast media; CNS—central nervous system; COX—cyclooxygenase, *n*—number.

**Table 9:** Characteristics of Patients with Fatal SRsAs in Relation to Drug Categories.

Causative Group of Drugs	Age		Females % (*n*)
	Mean (SD)	Min; Max	
ABs	48.2 (16.1)	15 months; 85 years	53.8 (50)
LAs	39.4 (14.4)	5 months; 68 years	55.1 (16)
CV drugs	62.6 (10.6)	48 years; 86 years	30.8 (4)
ICCM	74.0 (8.2)	65 years; 86 years	62.5 (5)
NMBAs	37.0 (12.7)	22 years; 61 years	75.0 (6)
COX inhibitors	50.7 (0.4)	50 years; 51 years	75.0 (3)
CNS-active drugs	45.6 (7.7)	32 years; 55 years	60.0 (3)
Other drugs	51.4 (15.2)	1 month; 80 years	57.8 (41)

ABs—antibacterials; LAs—local anesthetics; CV—cardiovascular; ICCM—iodine-containing contrast media; NMBAs—neuromuscular blocking agents; COX—cyclooxygenase; CNS—central nervous system; SD—standard deviation; *n*—number.

**Table 10:** ABs involved in Anaphylaxis in Children.

ABs	*n* (Total—57)	%
*Beta-lactams*	*50*	*87.7*
Ceftriaxone	29	50.9
Cefotaxime	7	12.3
Cefazolin	5	8.8
Ampicillin sulbactam	4	7.0
Cefepime	2	3.5
Cefoperazone sulbactam	1	1.8
Meropenem	1	1.8
Amoxicillin clavulanate	1	1.8
*Other*	*7*	*12.3*
Vancomycin	4	7.0
Metronidazole	2	3.5
Linezolid	1	1.8

ABs—antibacterials; *n*—number.

**Table 11:** LAs involved in Anaphylaxis in Children.

LAs	*n* (Total—17)	%
Lidocaine	9	52.9
Articaine	6	35.3
Mepivacaine	2	11.8

LAs—local anesthetics; *n*—number.

**Table 12:** Drugs involved in SRsAs in the elderly.

Drug	*n* (Total—65)	%
*ABs*	*26*	*40.0*
Ceftriaxone	23	35.4
Amoxicillin clavulanate	2	3.1
Cefixime	1	1.5
*CV drugs*	*13*	*20.0*
Antiarrhythmic (Amiodarone)	3	4.6
Unfractionated heparin	2	3.1
Beta-blockers (Bisoprolol)	2	3.1
Calcium Channels Blockers	3	4.6
Amlodipine	2	3.1
Nifedipine	1	1.5
Diuretics	2	3.1
Furosemide	1	1.5
Hydrochlorothiazide	1	1.5
Thrombolytic agent (Alteplase)	1	1.5
*ICCM*	*8*	*12.3*
Iopromide	4	6.2
Iohexol	3	4.6
Iomeprol	1	1.5
*COX inhibitors*	*5*	*7.7*
Diclofenac	3	4.6
Lornoxicam	1	1.5
Metamizole	1	1.5
*NMBAs*	*4*	*6.2*
Rocuronium	2	3.1
Atracurium	1	1.5
Cisatracurium	1	1.5
*LAs*	*2*	*3.1*
Bupivacaine	1	1.5
Procaine	1	1.5
*Other drugs*	*7*	*10.8*
Prednisolone	1	1.5
Iron formulations	1	1.5
Ethyl-methyl-hydroxypyridine succinate	1	1.5
Venlafaxine	1	1.5
Oxaliplatin	1	1.5
Cisplatin	1	1.5
Pentoxifylline	1	1.5

ABs—antibacterials; CV—cardiovascular; ICCM—iodine-containing contrast media; COX—cyclooxygenase; NMBAs—neuromuscular blocking agents; LAs—local anesthetics; *n*—number.

## Data Availability

Data were gained from https://newmimn.roszdravnadzor.gov.ru/ (accessed on 21 June 2023).
